# Two Years’ Experience with a Dental Program

**Published:** 1922-07

**Authors:** John C. Gebhart

**Affiliations:** Director, Department of Social Welfare, New York Association for Improving the Condition of the Poor, New York, N. Y.


### TWO YEARS' EXPERIENCE WITH A DENTAL PROGRAM

BY JOHN C. GEBHART

Director, Department of Social Welfare, New York Association for Improving the Condition of the Poor, New York, N. Y.

To be successful a preventive dental program must place its emphasis on the younger children, it must follow these children through from a sufficient period to secure positive results and it must be prophylactic and educational in spirit rather than curative and reparative. For over two years the Association for Improving the Condition of the Poor has been carrying out such a program with the co-operation of the health and education departments of the city in a congested district of 35,000 population.

Before this intensive program was undertaken two dental clinics for school children were operating in this district; one at public school No. 21 with a dentist on half time and one at the Italian school of the Children's Aid Society with a dentist three mornings each week. The other schools in the district, public school No. 106, public school No. 130 and St. Patrick's Parochial School were receiving no dental service. Our procedure has been to supplement existing facilities and to supply them where entirely lacking in order to provide a 100 per cent. program for the children of this area. The work will be carried on for a sufficient period to secure tangible results and to perfect methods of procedure which will lead eventually to the community itself assuming the burden of carrying on the work on a permanent basis.

The following steps have been laid down by the Dental Advisory Committee in the order of their importance:

1. Prophylactic cleanings at least once a year, and twice a year if possible, for all children in the first five grades of school.

2. Extraction of unsavable and diseased teeth, to put the mouth in a hygienic condition.

3. Prophylactic fillings (to prevent decay) in all first permanent molars and reparative fillings where needed in first molars.

4. Nitrate of silver treatment to arrest decay in deciduous teeth with fillings in those deciduous teeth which are retained longest.

The work was begun October 1, 1919, with one dental hygienist who made prophylactic cleanings in one of the public schools and instructed the children in oral hygiene. Beside a supervising dentist on part time we have now a staff of three operating dentists, one on full time, the other two on part time, and three full-time hygienists. Each year the Columbia School of Oral Hygiene has put a staff of from ten to fifteen undergraduate students for field practice in the schools of this district.

During the first year, October 1, 1919, to October 1, 1920, our records showed that 1748 children were given some sort of dental treatment by our own staff. The chief operations were 1691 fillings, 1103 extractions and 1466 prophylactic cleanings. The students of the Columbia School of Oral Hygiene provided about 1500 prophylactic cleanings.

A careful analysis was made of the record cards of children treated between January 1, 1920, and July 1, 1921. The report does not give us the complete picture of what was accomplished during this period, because our record-keeping system had not been perfected until the latter part of the period and several hundred cases were not accounted for.

The following (Table I) indicates the number of children treated in each school and the amount of work done:

TABLE I.

*Total Children Treated to July 1, 1921.*

| | Public School No. 106. | | Public School No. 21. | | Public School No. 130. | | St. Patrick's School. | | Total All Schools. | |
|---|---|---|---|---|---|---|---|---|---|---|
| | No. Children Treated. | No. of Cleanings. | No. Children Treated. | No. of Cleanings. | No. Children Treated. | No. of Cleanings. | No. Children Treated. | No. of Cleanings. | No. Children Treated. | No. of Cleanings. |
| No Cleaning .... | 19 | .... | .... | .... | 24 | .... | 12 | .... | 55 | |
| One Cleaning .... | 439 | 439 | 767 | 767 | 609 | 609 | 795 | 795 | 2,610 | 2,610 |
| Two Cleanings ... | 329 | 658 | 356 | 712 | 11 | 22 | 256 | 512 | 952 | 1,904 |
| Three Cleanings .. | 100 | 300 | 85 | 255 | .... | .... | 100 | 300 | 285 | 855 |
| Four Cleanings .. | 1 | 4 | 2 | 8 | .... | .... | 5 | 20 | 8 | 32 |
| Five Cleanings .. | ... | .... | .... | .... | .... | .... | 1 | 5 | 1 | 5 |
| Total ....... | 888 | 1,401 | 1,210 | 1,742 | 644 | 631 | 1,169 | 1,632 | 3,911 | 5,406 |
| Total completed for dentistry .. | 781 | .... | 424* | .... | 28 | .... | 285 | .... | 1,518 | |
| | 88% | .... | 35% | .... | 4% | .... | 24% | .... | 38% | |

* Dentistry performed by the Health Department of New York City. Cleanings by the Dental Hygienist of the Association for Improving the Condition of the Poor.

The most complete work was accomplished at public school No. 106, and probably the most complete piece of work of any public school in the country. In this school, as Table I shows, 88 per cent. of the children had their dentistry completed. Of the entire group treated, 430, or 48 per cent. of the children, had two or more cleanings.

In order to determine, if possible, the improvement in mouth conditions with each successive cleaning, the children were divided into groups of: (1) Those who had one cleaning only, (2) those who had two cleanings, and (3) those with three or more cleanings. There is a steady decline with each cleaning in the number of cavities found and an increase in the number of children with no cavities. This is clearly brought out in Table II.

TABLE II.

*Summary of Examinations of Each Group.—Public School No. 106.*

| | First Cleaning. | | | | | | Second Cleaning. | | | | | Third Cleaning. | | | | |
|---|---|---|---|---|---|---|---|---|---|---|---|---|---|---|---|---|
| | No. Children Treated. | No. Cavities, Deciduous Teeth. | No. Cavities, Permanent Teeth. | Cavities per Child. | No Cavities. | Percentage. | No. Cavities, Deciduous Teeth. | No. Cavities, Permanent Teeth. | Cavities per Child. | No Cavities. | Percentage. | No. Cavities, Deciduous Teeth. | No. Cavities, Permanent Teeth. | Cavities per Child. | No Cavities. | Percentage. |
| Group A (One Cleaning). . | 439 | 1,364 | 1,284 | 6. | 38 | 8.6 | | | | | | | | | | |
| Group B (Two Cleanings). | 329 | 1,052 | 975 | 6.1 | 26 | 8.2 | 592 | 457 | 3.2 | 115 | 35 | | | | | |
| Group C (Three Cleanings). | 101 | 367 | 349 | 7.1 | 5 | 5.0 | 233 | 272 | 5.0 | 13 | 13 | 147 | 118 | 2.6 | 42 | 42 |
| Total. ........ | 869 | 2.783 | 2.608 | 6.2 | 69 | 8.6 | 825 | 729 | 3.6 | 128 | 30 | 147 | 118 | 2.6 | 42 | 42 |

Dr. Kinney makes each year a classification of the dental defects found in the children of each grade by means of a group examination of the entire school. The comparison of defects found in October of last year will also serve to indicate the progress made with the entire group under care.

The relative increase and decrease of various items is best shown by comparing the various items in both surveys. The classifications in the 1921 survey have been combined in the summary to make them comparable to those of 1920, as shown in Table III.

TABLE III.

| | No. Children Examined. | Extractions Needed. | | | | Fillings Needed. | | | | | | No. Attention Needed. | |
|---|---|---|---|---|---|---|---|---|---|---|---|---|---|
| | | Abscessed. | | Others. | | Urgent. | | Medium. | | Mild. | | | |
| | | No. | Per. cent. | No. | Per. cent. | No. | Per. cent. | No. | Per. cent. | No. | Per. cent. | No. | Per. cent. |
| 1920 | 840 | 118 | 14.5 | 266 | 31.7 | 432 | 51.4 | 102 | 12.1 | 183 | 21.8 | 90 | 10.7 |
| 1921 | 821 | 258 | 31.1 | 60 | 7.2 | 156 | 19.2 | 17 | 2.0 | 188 | 22.7 | 156 | 18.8 |

There is thus a marked decrease in the fillings needed except for a slight increase in the group classified as "mild." There is also an increase in the number of children needing no attention. While there is a gross decrease in the children needing extractions there is actually an increase over last year in those with no abscessed teeth. This, I think, may be explained by the fact that the study was made at a later date last year, after many of the worst conditions had been cleared up. Reference to the monthly reports bears this out. Whereas last year 85 per cent. of the children were in need of fillings, only 44 per cent. required fillings this year; 38 per cent. needed extractions as against 46 per cent. last year; but 18.8 per cent. were free from dental defects as against 10.7 per cent. last year. Since the survey was made last year, two or three months after the work had been under way, it is safe to say that the amount of work to be done has been reduced 50 per cent. over last year.

A detailed monthly report of the work has been kept since October 1, 1920. During the period certain items have been added, so that a complete report of the year for each item is not possible. The following summary gives the total for the entire year or for the portion of the year for which the data are available:

SUMMARY OF WORK DONE

| | Total for year |
|---|---|
| Fillings: | |
| Preparation of cavities | 7,067 |
| Treatments | 748 |
| Fillings in permanent teeth | 6,269 |
| Fillings in deciduous teeth | 1,108 |
| Nitrate of silver—deciduous teeth | 628 |
| Total operations (preparation, treatment, fillings) | 16,662 |
| First molars filled (10 mos. only) | 2,197 |
| No. hours (for fillings, etc.) (9 mos. only) | 2,514 |
| Extractions: | |
| No. patients | 601 |
| No. extractions—deciduous teeth | 1,036 |
| No. extractions—permanent teeth | 157 |
| Total extractions | 1,191 |

| | |
|---|---|
| Prophylactic cleanings | 4,574 |
| No. hours—hygienists (7 mos. only) | 1,631 |
| No. children without cavities | 375 |
| Percentage of children without cavities | 10 |
| Total operations | 21,585 |
| Prophylactic cleanings by Columbia School of Oral Hygiene | 1,699 |
| Grand total operations | 23,284 |

We are now able to reach tentative conclusions as to the time required to complete various operations and the cost of such service. If we divide the total completed fillings for nine months, 7,493, by the hours spent by the dental operators, 2,513, we get about 3 fillings per hour, or to be exact, 20.1 minutes per filling. We have disregarded the time taken for nitrate of silver treatments, which were also done on the operator's time, because we have no report on the time required for this work. If we allow five minutes for a nitrate of silver treatment 2.8 fillings are completed per hour, or about 22 minutes per filling.

For seven months we have had a check on the time required for a prophylactic cleaning. It took 1,631 hours to make 2,360 prophylactic cleanings, *i. e.,* 1.4 cleanings per hour, or .73 hour per cleaning. In other words, it takes 44 minutes to give a child a prophylactic cleaning.

We are now able to arrive at a tentative figure of the per capita cost of the work. During the year 4,574 cleanings were made by the Association for Improving the Condition of the Poor staff and 1,699 by the Columbia staff, a total of 6,273 cleanings. Our procedure is to give two cleanings per child so that we may say that 3,136 children were reached through our efforts at least for prophylactic cleaning. Of these approximately 1,500 received corrective dentistry as well. The total cost for looking after 3,136 children was $12,961.67, or $4.13 per child per year. This includes several hundred dollars for permanent equipment which, if deducted, brings the cost to about $4.00 per child.

The cost per cleaning, allowing for the pay during vacation for each hygienist, is $0.66 per cleaning. The cost per completed filling is approximately $0.64, again allowing for pay for the dentist during the vacation period. In arriving at the cost of filling we have divided the total salaries of dental operators by the number of fillings. Some time, however, was given to nitrate of silver treatments and a little to extractions. If we were able to make the proper reductions for this the cost per filling would be a little in excess of this, probably $0.70 per filling.

The bulk of the extractions are made by the supervisor, Dr. Kinney, in addition to her other duties. We are, therefore, unable to determine the cost of extraction. For the future, however, we shall record the exact time put in on extractions by the supervisor in order to arrive at the cost of this service.

The per capita cost will gradually be reduced for operative work at least with each succeeding year. As we have pointed out above the amount of work needed to be done in public school No. 106 has been reduced 50 per cent. With each year there will be a slight reduction until we reach an irreducible minimum of work to be done.

This is the first attempt to determine the per capita cost of this type of service. Dr. Alfred C. Fones, of Bridgeport, Conn., states in *Dental Cosmos*, October 1921, the amount of money appropriated for the Bridgeport program to be $40,000 for 22,618. This means a per capita cost of $1.77 for a program given exclusively to prophylactic cleanings and fillings in first molars. We shall soon be able to determine fairly accurately the cost of a cleaning, a filling and extraction and the amount to be allowed for supervision, supplies and depreciation of equipment. We shall then be able to tell the exact cost of doing a given amount of work for any particular group. Such information is greatly needed by different agencies, both public and private, who are planning dental programs.—*Dental Cosmos.*

